# Supplementary material for: Prevalence of glucose-6-phosphate dehydrogenase deficiency (G6PDd), CareStart qualitative rapid diagnostic test performance, and genetic variants in two malaria-endemic areas in Sudan
Source: PLoS Negl Trop Dis. 2021 Oct 26;15(10):e0009720. doi: 10.1371/journal.pntd.0009720 (PMC8547650; doi:10.1371/journal.pntd.0009720)
Supplement: S1 Table — (DOCX) [file pntd.0009720.s001.docx]

**S1 Table. Detailed population characteristics, New Halfa and Khartoum, Sudan 2015. (N=491)**

| Characteristic | Value | n (% of total) |
| --- | --- | --- |
| Linguistic group | Nilo-Saharan | 163 (33.2 %) |
|  | Afro-Asiatic | 309 (62.9%) |
|  | Missing | 19 (3.9%) |
| Occupation | Other | 322 (65.6%) |
|  | Worker | 78 (15.9%) |
|  | Farmer | 49 (10.0%) |
|  | Missing | 42 (8.6%) |
| Education | Illiterate | 136 (27.7 %) |
|  | Primary/intermediate school | 151 (30.8%) |
|  | Other | 94 (19.1%) |
|  | Secondary school | 88 (17.9%) |
|  | Missing | 22 (4.5 %) |
| Pallor | No | 414 (84.3%) |
|  | Yes | 43 (8.8%) |
|  | Missing | 34 (6.9 %) |
| Jaundice- yellow sclera | No | 465 (94.7%) |
|  | Yes | 21 (4.3%) |
|  | Missing | 5 (1.0%) |
| Jaundice- yellow eyes | No | 442 (90.0%) |
|  | Yes | 43 (8.8%) |
|  | Missing | 6 (1.2%) |
| Jaundice- yellow skin | No | 474 (96.5%) |
|  | Yes | 11 (2.2%) |
|  | Missing | 6 (1.2%) |
| Hematuria | No | 408 (83.1%) |
|  | Yes | 69 (14.1%) |
|  | Missing | 14 (2.9%) |
| Any heart palpitations | No | 387 (77.0%) |
|  | Yes | 107 (21.8%) |
|  | Missing | 6 (1.2%) |
| Fatigue | No | 222 (45.2%) |
|  | Yes | 267 (54.4%) |
|  | Missing | 2 (0.4%) |
| Any antibiotics, prior 2 weeks | No | 367 (74.7%) |
|  | Yes | 117 (23.8%) |
|  | Missing | 2 (0.4%) |
| Any malaria treatment, prior 2 weeks | No | 392 (79.8%) |
|  | Yes | 95 (19.3%) |
|  | Missing | 7 (0.9%) |
| Able to eat fava beans | No | 26 (5.3%) |
|  | Yes | 459 (93.5%) |
|  | Missing | 6 (1.2%) |
| Any family history of anemia drugs | No | 474 (96.5%) |
|  | Yes | 11 (2.2%) |
|  | Missing | 6 (1.2%) |
| Any family history of bleeding disorders | No | 443 (90.2%) |
|  | Yes | 44 (9.0%) |
|  | Missing | 4 (0.8%) |
| Any family history of anemia | No | 461 (93.9%) |
|  | Yes | 28 (5.7%) |
|  | Missing | 2 (0.4%) |
| Any anemia drug usage, prior 2 weeks | No | 470 (95.7%) |
|  | Yes | 8 (1.6%) |
|  | Missing | 13 (2.6%) |
| Recent blood transfusion, prior 2 weeks | No | 470 (95.7%) |
|  | Yes | 18 (3.7%) |
|  | Missing | 3 (0.6%) |
