# Supplementary material for: Prevalence of glucose-6-phosphate dehydrogenase deficiency (G6PDd), CareStart qualitative rapid diagnostic test performance, and genetic variants in two malaria-endemic areas in Sudan
Source: PLoS Negl Trop Dis. 2021 Oct 26;15(10):e0009720. doi: 10.1371/journal.pntd.0009720 (PMC8547650; doi:10.1371/journal.pntd.0009720)
Supplement: S2 Table — (DOCX) [file pntd.0009720.s002.docx]

**S2 Table. Multivariable risk factors for Glucose-6-phosphate-dehydrogenase deficiency, Sudan (Deficiency defined as activity less than 30% of adjusted male median (AMM).
(N=490; imputed dataset).**

| Covariate | Adjusted Odds Ratio | 95% CI | p value |
| --- | --- | --- | --- |
| Gender |  |  |  |
| Female | Ref. | - |  |
| Male | 0.47 | 0.20 to 1.11 | 0.089 |
| Study site |  |  |  |
| New Halfa | Ref. | - |  |
| Khartoum | 0.55 | 0.13 to 2.40 | 0.425 |
| Data source |  |  |  |
| Private clinic | Ref. | - |  |
| Hospital | 0.38 | 0.13 to 1.11 | 0.078 |
| Ethnicity |  |  |  |
| Arab | Ref. |  |  |
| Nuba | 0.30 | 0.03 to 2.79 | 0.290 |
| Nubian | 0.35 | 0.04 to 2.9 | 0.330 |
| Darforian | 1.70 | 0.53 to 5.4 | 0.375 |
| Bbeja | 0.81 | 0.15 to 4.43 | 0.812 |
| Other | 0.46 | 0.05 to 3.9 | 0.473 |
| Occupation |  |  |  |
| Other | Ref. |  |  |
| Worker | 2.00 | 0.56 to 7.1 | 0.283 |
| Farmer | 1.11 | 0.25 to 5.0 | 0.891 |
| Antibiotics, prior 2 weeks |  |  |  |
| No | Ref. |  |  |
| Yes | 1.49 | 0.65 to 3.37 | 0.344 |
| Malaria drug, prior 2 weeks |  |  |  |
| No | Ref. |  |  |
| Yes | 1.57 | 0.63 to 3.90 | 0.327 |
| Family history, bleeding disorders |  |  |  |
| No | Ref. |  |  |
| Yes | 1.33 | 0.41 to 4.35 | 0.638 |
| Age, years | 1.02 | 0.995 to 1.05 | 0.101 |
| Weight, kg | 0.97 | 0.95 to 0.99 | 0.010 |
