# Supplementary material for: Prevalence of glucose-6-phosphate dehydrogenase deficiency (G6PDd), CareStart qualitative rapid diagnostic test performance, and genetic variants in two malaria-endemic areas in Sudan
Source: PLoS Negl Trop Dis. 2021 Oct 26;15(10):e0009720. doi: 10.1371/journal.pntd.0009720 (PMC8547650; doi:10.1371/journal.pntd.0009720)
Supplement: Attachment — Submitted filename: PNTD-D-21-00031_reviewer_comments.docx [file pntd.0009720.s003.docx]

Albsheer and colleagues report valuable findings on the prevalence and genetic variants of G6PD deficiency within Sudan, as well as the performance of the CareStart qualitative point-of-care (POC) G6PDd RDT. Their survey covered 557 individuals across two malaria-endemic sites in Sudan, employing UV spectrophotometry, a novel POC assay and PCR techniques to survey the prevalence and genetic variants of G6PDd present. The authors report a low-to-moderate G6PD prevalence, a predominance of G6PD A variants among deficient individuals, and moderate diagnostic performance of the CareStart POC assay, particularly limited among females. This is an important and timely paper that provides important evidence towards supporting policies targeting *Plasmodium vivax* in Sudan and I commend the authors on their important work.

I have a few key comments on how some aspects of the methodology and results are presented that I believe should be clarified and addressed prior to publication, as well as a number of more minor typographical/stylistic comments/corrections below.

**Key points:**

1. The first area requiring clarification is with regards to the reference assay used in this study. The authors mention that gold standard spectrophotometry was used, however given the sensitivity of this assay to a host of factors (e.g. temperature, sample storage/transportation) and the importance of the reference assay for assessing diagnostic performance it may be worth elaborating on a number of key points here to reassure readers e.g.:
   - Which test kit (Trinity, Randox, Pointe Scientific) was used?
   - At what temperature and wavelength were samples measured? Did this involve a temperature-controlled spectrophotometer?
   - What was the typical delay between sampling and measurement? (e.g. within 2, 4, 6, 10hours?)
   - **Importantly**: Were manufacturer-provided control reagents used to ensure readings fell within the expected range? (e.g. measurement of G6PD deficient, intermediate/normal controls).
   - Related to this: I note that a number of extreme (>25 or even >30 U/g Hb) G6PD activity readings were identified. In my experience this can represent an issue with the readings for that particular sample, and it is not uncommon to censor or re-test readings above a maximum threshold. The authors may wish to briefly mention in their results/methods how extreme values were handled.
2. The second area that I believe requires clarification to aid in its interpretation is the reporting of genotyping results. It is very important that G6PD genotyping findings are reported in terms of which genetic variants you have looked for. Here the authors have tested for polymorphisms at the 202/376 loci, but it does not appear that they have tested for other mutations at other sites/exons – e.g. Mediterranean. Therefore, these findings are very useful in describing heterogeneity at the loci observed, but don’t necessarily rule out the presence of alternative variants.

As such I suggest the wording of the discussion and results section be tweaked to reflect this. Importantly, I strongly suggest the addition of a sentence to the effect of ‘Of the 22 subjects with G6PD deficiency identified using spectrophotometry, X were found to have a mutation consistent with the A genotype (XX hemi/homozygous, XX heterozygous). No mutation was identified at the 376/202 loci for the remaining XX deficient individuals’. It is slightly unclear currently whether all of the 22 individuals found to be deficient had one of the underlying combinations of mutations consistent with an ‘A’ genotype or whether only some did, along with a number of the G6PD normal individuals. As this genotype is typically associated with less severe deficiency than A- (both mutations), the fact that the authors identify very severe deficiency in this cohort is an important finding. Finally, I believe the authors should address in their discussion the possibility of the presence of other variants (e.g. Mediterranean, ‘G6PD Khartoum’) in this cohort.

3. The final area that may benefit from a small amount of additional detail is in the reporting of the POC results. A key factor influencing the performance of any POC test in the field will be the ‘usability’ of the assay for fieldworkers. Do the authors have any insights they could report on this front? E.g. Were indeterminate results ever/often encountered?

**Additional comments/Other items to address:**

*Abstract & Introduction:*

- In the abstract, consider specifying that the study used a *qualitative* CareStart RDT to avoid possible confusion with the quantitative POC CareStart biosensor at first glance.

*Methods*:

- For consistency with the literature and original work defining the metric, I would suggest the authors refer to their ‘normal’ G6PD activity definition as the ‘Adjusted Male Median (AMM)’ when describing the population-level cutoffs, both in the methods and throughout the results.
- When describing the sequencing methods used to confirm genotyping I would suggest elaborating on the following:
  - Were all exons sequenced or just exons 4&5 as mentioned above? (see discussion above regarding possibility of other mutations from other exons in this population)
  - How were the subset of 60 samples for sequencing chosen? Based on indeterminate genotyping from PCR-RFLP or random selection from all samples?

*Results*:

- I suggest the authors briefly report on missing data towards the start of the results section – e.g. for key variables from the regression analysis, how many individuals had missing data, did individuals with missing data differ significantly from those without missing data? (e.g. based on G6PD status/study site)
- The final sentence of the biochemical testing section appears unfinished: ‘The population-specific trimmed male median value marked (3.8 U/g Hb across both sites; 3.2U/g Hb for New Halfa and 4.3 U/g Hb for Khartoum) (Table 3).’
- In the section on POC assay results, the authors state that: ‘Of these tests, 553 results were available for analysis’. If available, it would be advantageous to mention why the remaining 4 results were unavailable – e.g. were these indeterminate results for example?
- In the section on predictors of G6PDd: Note that an odds ratio cannot be interpreted directly in terms of ‘risk’. Instead, this should read: ‘This indicates that each increasing kg lowers the **odds** of being G6PD deficient (activity <3.8 U/g Hg), by approximately 3% (See Supplemental data; table S1)’.
- In the section on genotyping results:
  - It may be worth briefly mentioning whether any of the individuals for whom genotyping results were not available/PCR was unsuccessful were G6PD deficient.
  - I would recommend adding a sentence to the start of this section reiterating the total number of deficient found, how many of these had successful PCR/RFLP results, and of these how many were assigned a g6pd-deficient genotype (A/A-) versus how many were enzymatically deficient but for which a deficient mutation was not yet identified (see above).
  - It may be advantageous to be slightly more precise when describing the statistical comparison between sites at the end of this section: are the authors comparing here the frequency of a specific mutation? Or all identified genotypes at a particular locus?

*Discussion*:

- The authors state: “A recent study conducted in Northern Sudan reported the polymorphic genetic variants of the enzyme [5]”. It would be beneficial to elaborate – which variants did they look for? Which variants did they find? What was the relative frequency of identified variants?
- I would suggest rewording this sentence: “The limited use of the test in females is due to the fact that the heterozygous females produced heterogeneous levels of the enzyme ranging from low to almost normal level due to the enzyme activity is intermediate among heterozygous females”.

Heterogeneity in heterozygous females is not due to difference in the *amount* of enzyme produced between individuals, but due to random inactivation of one x-chromosome during embryogenesis, resulting in different proportions of deficient and normal red blood cells in different heterozygotes.

- Note unfinished brackets represented by ‘XX’ in the sentence “While G6PD AB variant was present in 1.1% (XX/557)”.
- While not a major aim of this study, the authors’ discussion of their findings regarding body weight as a predictor of G6PDd would benefit from a little more depth. Do the authors believe this is a true causal relationship? Perhaps they can comment briefly in the discussion on the confounding they already identified (i.e. in the relationship between study site and G6PDd due to body weight.) and dig into this a little deeper. What other possible explanations might there be for this finding? (e.g. other possible confounding variables, slight inconsistencies in laboratory procedures between sites). How might missing data (and/or imputation of missing variables) have influenced this finding?

*Figures and tables:*

- Table 1:
  - To ease interpretation, I would suggest moving most of the clinical variables from the second half of this table to a separate table or supplementary table and keeping just the core demographic variables plus the most important variables likely to impact upon G6PD status – e.g. malaria infection, blood transfusion, anemia, history of jaundice/favism
  - Additionally: the dis-aggregated numbers for multiple sub-sections here do not sum to **557**. The authors should consider adding an ‘other’, ‘unknown’ or ‘missing’ category for participants who don’t fit any of the listed categories:
    - Data source: 125+426 = **551**
    - Linguistic group: 173+364 =**537**
    - Occupation: 370+89+53=**512**
    - Education: 147+178+109+101=**535**
    - Anemia: 525+31 = **556**
    - A number of the remaining sections are similar – I suggest double checking each of these.
  - Finally, I suggest the authors add units for G6PD by spectrophotometry – presumably U/g Hb.
- Table 3 & associated results text: I would report either a mean or a median for G6PD activity values, not both. In this case perhaps the median is most appropriate
- Table 4: I suggest adding specificity estimates to the summaries below this table for completeness sake.
- Table 5: Its very difficult to present these data in an easily interpreted manor, so I commend the authors for a good job of presenting this in an easy-to-read table. To aid interpretation further, by reducing ambiguity, I would recommend extending this table to include all possible combinations – e.g. explicitly reporting that 0 hemizygous A- males or homozygous A- females, were observed in either site. E.g. (additions in red).

| Sex | Genotype | Phenotype Label | Interpretation | New Halfa | Khartoum |
| --- | --- | --- | --- | --- | --- |
| M | 202G/376A | B | Hemizygous WT |  |  |
|  | 202G/376G | A | Hemizygous A |  |  |
|  | 202A/376A |  |  |  |  |
|  | 202A/376G | A- | Hemizygous A- |  |  |
| F | 202G, 202G/376A, 376A | B/B | Homozygous WT |  |  |
|  | 202A, 202G/376A, 376A  202G, 202G/376G, 376A | A/B | Heterozygous A |  |  |
|  | 202G, 202G/376G, 376G  202A, 202A/376A, 376A | A/A | Homozygous A |  |  |
|  | 202A, 202G/376G, 376A | A-/B | Heterozygous A- |  |  |
|  | 202A, 202A/ 376G, 376G | A-/A- | Homozygous A- |  |  |

- Table 6:
  - It is unclear why different categories are presented in this table compared with figure 4 – it would be easier to compare if same groupings were used in both.
  - It is unclear what ‘all other’ refers to here – are these individuals that **do** have a variant at 376/202 (this table title suggests only variant individuals are included) but its unclear which specific mutation is present?
  - Again, I don’t think the mean is necessary here, id suggest the authors only report the median.
  - It may be beneficial to add here ‘translations’ of the genotypes for easier interpretation, e.g. a column that reads: ‘B’ for normal males, ‘A/A’ for homozygous A females etc.
- Figures 1-3: I suggest adding units to the x axis – U/g Hb.
- Figure 4: It is unclear what ‘other’ refers to here, perhaps a footnote explaining this would be useful. Are these individuals who could not be genotyped at these loci?

*Minor typographical/textual Comments:*

- Title – Likely a typo, but I would recommend adding a space between phosphate and dehydrogenase in the title.
- Abstract: ‘Such evidence based data will guide the expansion of primaquine for P. vivax infections radical cure.’ Consider changing to ‘… radical cure of *P. vivax* infections’.
