# Supplementary material for: Prevalence of glucose-6-phosphate dehydrogenase deficiency (G6PDd), CareStart qualitative rapid diagnostic test performance, and genetic variants in two malaria-endemic areas in Sudan
Source: PLoS Negl Trop Dis. 2021 Oct 26;15(10):e0009720. doi: 10.1371/journal.pntd.0009720 (PMC8547650; doi:10.1371/journal.pntd.0009720)
Supplement: Attachment — Submitted filename: PNTD-D-21-00031_responses to reviewers.docx [file pntd.0009720.s004.docx]

**Responses to the reviewers**

\## General response from authors

We greatly appreciate the detailed and thoughtful comments from both reviewers. To address these, we have updated all analyses; expanded the methods and refined the discussion; and have copy-edited throughout for clarity. We have also added tafenoquine-related breakpoints where appropriate. All changes have been tracked in the “TRACKED.docx” file.

**Review #1**

Albsheer and colleagues report valuable findings on the prevalence and genetic variants of G6PD deficiency within Sudan, as well as the performance of the CareStart qualitative point-of-care (POC) G6PDd RDT. Their survey covered 557 individuals across two malaria-endemic sites in Sudan, employing UV spectrophotometry, a novel POC assay and PCR techniques to survey the prevalence and genetic variants of G6PDd present. The authors report a low-to-moderate G6PD prevalence, a predominance of G6PD A variants among deficient individuals, and moderate diagnostic performance of the CareStart POC assay, particularly limited among females. This is an important and timely paper that provides important evidence towards supporting policies targeting *Plasmodium vivax* in Sudan and I commend the authors on their important work.

I have a few key comments on how some aspects of the methodology and results are presented that I believe should be clarified and addressed prior to publication, as well as a number of more minor typographical/stylistic comments/corrections below.

**Key points:**

1. The first area requiring clarification is with regards to the reference assay used in this study. The authors mention that gold standard spectrophotometry was used, however given the sensitivity of this assay to a host of factors (e.g. temperature, sample storage/transportation) and the importance of the reference assay for assessing diagnostic performance it may be worth elaborating on a number of key points here to reassure readers e.g.:
   - Which test kit (Trinity, Randox, Pointe Scientific) was used?

\## Response from authors: This has been clarified in the text; # SPINREACT™, Spain

- At what temperature and wavelength were samples measured? Did this involve a temperature-controlled spectrophotometer?

\## Response from authors: This has been clarified in the text; room temperature (25˚C)

- What was the typical delay between sampling and measurement? (e.g. within 2, 4, 6, 10hours?)

\## Response from authors: This has been clarified in the text; done within 30 minutes after collection

- **Importantly**: Were manufacturer-provided control reagents used to ensure readings fell within the expected range? (e.g. measurement of G6PD deficient, intermediate/normal controls).

\## Response from authors: This has been clarified in the text; G6PD normal and deficient controls (Ref: 100 252 0, SPINREACT, Spain), were run daily prior to sample measurement.

- Related to this: I note that a number of extreme (>25 or even >30 U/g Hb) G6PD activity readings were identified. In my experience this can represent an issue with the readings for that particular sample, and it is not uncommon to censor or re-test readings above a maximum threshold. The authors may wish to briefly mention in their results/methods how extreme values were handled.

** Response from authors. These extreme values were not re-analyzed during the lab work, but were removed from analysis.

2. The second area that I believe requires clarification to aid in its interpretation is the reporting of genotyping results. It is very important that G6PD genotyping findings are reported in terms of which genetic variants you have looked for. Here the authors have tested for polymorphisms at the 202/376 loci, but it does not appear that they have tested for other mutations at other sites/exons – e.g. Mediterranean. Therefore, these findings are very useful in describing heterogeneity at the loci observed, but don’t necessarily rule out the presence of alternative variants.

As such I suggest the wording of the discussion and results section be tweaked to reflect this. Importantly, I strongly suggest the addition of a sentence to the effect of ‘Of the 22 subjects with G6PD deficiency identified using spectrophotometry, X were found to have a mutation consistent with the A genotype (XX hemi/homozygous, XX heterozygous). No mutation was identified at the 376/202 loci for the remaining XX deficient individuals’. It is slightly unclear currently whether all of the 22 individuals found to be deficient had one of the underlying combinations of mutations consistent with an ‘A’ genotype or whether only some did, along with a number of the G6PD normal individuals. As this genotype is typically associated with less severe deficiency than A- (both mutations), the fact that the authors identify very severe deficiency in this cohort is an important finding. Finally, I believe the authors should address in their discussion the possibility of the presence of other variants (e.g. Mediterranean, ‘G6PD Khartoum’) in this cohort.

** Response from authors Thank you for these suggestions; we have incorporated all these changes in the results and discussion of the newest version.

3. The final area that may benefit from a small amount of additional detail is in the reporting of the POC results. A key factor influencing the performance of any POC test in the field will be the ‘usability’ of the assay for fieldworkers. Do the authors have any insights they could report on this front? E.g. Were indeterminate results ever/often encountered?

** Response from authors Thank you for this suggestion; we did not collect any usability data during these studies, so are unable to add any comments here. Regarding Carestart test and whether it provides intermediate results, it only provides either deficient or not deficient result.

**Additional comments/Other items to address:**

*Abstract & Introduction:*

- In the abstract, consider specifying that the study used a *qualitative* CareStart RDT to avoid possible confusion with the quantitative POC CareStart biosensor at first glance.

** Response from authors we have clarified this issue throughout the text to avoid any ambiguity.

*Methods*:

- For consistency with the literature and original work defining the metric, I would suggest the authors refer to their ‘normal’ G6PD activity definition as the ‘Adjusted Male Median (AMM)’ when describing the population-level cutoffs, both in the methods and throughout the results.
- When describing the sequencing methods used to confirm genotyping I would suggest elaborating on the following:
  - Were all exons sequenced or just exons 4&5 as mentioned above? (see discussion above regarding possibility of other mutations from other exons in this population)
  - How were the subset of 60 samples for sequencing chosen? Based on indeterminate genotyping from PCR-RFLP or random selection from all samples?

** Response from authors we have updated all mentions to include AMM.

We sequenced only exon 4 and 5 and that was added in the text.

A subset was chosen to represent different fragment length polymorphisms patterns. And a sentence is added in the text.

*Results*:

- I suggest the authors briefly report on missing data towards the start of the results section – e.g. for key variables from the regression analysis, how many individuals had missing data, did individuals with missing data differ significantly from those without missing data? (e.g. based on G6PD status/study site)

** Response from authors: We have expanded discussion of missing data throughout the text.

- The final sentence of the biochemical testing section appears unfinished: ‘The population-specific trimmed male median value marked (3.8 U/g Hb across both sites; 3.2U/g Hb for New Halfa and 4.3 U/g Hb for Khartoum) (Table 3).’

  ** Response from authors: This has been corrected.
- In the section on POC assay results, the authors state that: ‘Of these tests, 553 results were available for analysis’. If available, it would be advantageous to mention why the remaining 4 results were unavailable – e.g. were these indeterminate results for example?
- In the section on predictors of G6PDd: Note that an odds ratio cannot be interpreted directly in terms of ‘risk’. Instead, this should read: ‘This indicates that each increasing kg lowers the **odds** of being G6PD deficient (activity <3.8 U/g Hg), by approximately 3% (See Supplemental data; table S1)’.

** Response from authors we have revised the results and added details here to address these lapses and clarified discussion of the associations.

- In the section on genotyping results:
  - It may be worth briefly mentioning whether any of the individuals for whom genotyping results were not available/PCR was unsuccessful were G6PD deficient.
  - I would recommend adding a sentence to the start of this section reiterating the total number of deficient found, how many of these had successful PCR/RFLP results, and of these how many were assigned a g6pd-deficient genotype (A/A-) versus how many were enzymatically deficient but for which a deficient mutation was not yet identified (see above).

** Response from authors: All G6PD deficient individuals had available PCR and genotyping results and information was added in the text.

It may be advantageous to be slightly more precise when describing the statistical comparison between sites at the end of this section: are the authors comparing here the frequency of a specific mutation? Or all identified genotypes at a particular locus?

** Response from authors: This has been clarified in the text.

*Discussion*:

- The authors state: “A recent study conducted in Northern Sudan reported the polymorphic genetic variants of the enzyme [5]”. It would be beneficial to elaborate – which variants did they look for? Which variants did they find? What was the relative frequency of identified variants?

\## Response from authors: This has been clarified in the text.

- I would suggest rewording this sentence: “The limited use of the test in females is due to the fact that the heterozygous females produced heterogeneous levels of the enzyme ranging from low to almost normal level due to the enzyme activity is intermediate among heterozygous females”.

Heterogeneity in heterozygous females is not due to difference in the *amount* of enzyme produced between individuals, but due to random inactivation of one x-chromosome during embryogenesis, resulting in different proportions of deficient and normal red blood cells in different heterozygotes.

- Note unfinished brackets represented by ‘XX’ in the sentence “While G6PD AB variant was present in 1.1% (XX/557)”.

\## Response from authors: These issues have been revised and addressed.

- While not a major aim of this study, the authors’ discussion of their findings regarding body weight as a predictor of G6PDd would benefit from a little more depth. Do the authors believe this is a true causal relationship? Perhaps they can comment briefly in the discussion on the confounding they already identified (i.e. in the relationship between study site and G6PDd due to body weight.) and dig into this a little deeper. What other possible explanations might there be for this finding? (e.g. other possible confounding variables, slight inconsistencies in laboratory procedures between sites). How might missing data (and/or imputation of missing variables) have influenced this finding?

\## Response from authors: Discussion on this point has been expanded as is likely a spurious result.

*Figures and tables:*

- Table 1:
  - To ease interpretation, I would suggest moving most of the clinical variables from the second half of this table to a separate table or supplementary table and keeping just the core demographic variables plus the most important variables likely to impact upon G6PD status – e.g. malaria infection, blood transfusion, anemia, history of jaundice/favism

** Response from authors This important point has been addressed and all “extra” values added to the supplemental info.

- Additionally: the dis-aggregated numbers for multiple sub-sections here do not sum to **557**. The authors should consider adding an ‘other’, ‘unknown’ or ‘missing’ category for participants who don’t fit any of the listed categories:
  - Data source: 125+426 = **551**
  - Linguistic group: 173+364 =**537**
  - Occupation: 370+89+53=**512**
  - Education: 147+178+109+101=**535**
  - Anemia: 525+31 = **556**
  - A number of the remaining sections are similar – I suggest double checking each of these.

** Response from authors A “missing: category has been added to all variables to aid interpretation of these tables.

- Finally, I suggest the authors add units for G6PD by spectrophotometry – presumably U/g Hb.

** Response from authors Added throughout the text.

- Table 3 & associated results text: I would report either a mean or a median for G6PD activity values, not both. In this case perhaps the median is most appropriate

** Response from authors Mean values removed.

- Table 4: I suggest adding specificity estimates to the summaries below this table for completeness sake.

** Response from authors Added.

- Table 5: Its very difficult to present these data in an easily interpreted manor, so I commend the authors for a good job of presenting this in an easy-to-read table. To aid interpretation further, by reducing ambiguity, I would recommend extending this table to include all possible combinations – e.g. explicitly reporting that 0 hemizygous A- males or homozygous A- females, were observed in either site. E.g. (additions in red).

| Sex | Genotype | Phenotype Label | Interpretation | New Halfa | Khartoum |
| --- | --- | --- | --- | --- | --- |
| M | 202G/376A | B | Hemizygous WT |  |  |
|  | 202G/376G | A | Hemizygous A |  |  |
|  | 202A/376A |  |  |  |  |
|  | 202A/376G | A- | Hemizygous A- |  |  |
| F | 202G, 202G/376A, 376A | B/B | Homozygous WT |  |  |
|  | 202A, 202G/376A, 376A  202G, 202G/376G, 376A | A/B | Heterozygous A |  |  |
|  | 202G, 202G/376G, 376G  202A, 202A/376A, 376A | A/A | Homozygous A |  |  |
|  | 202A, 202G/376G, 376A | A-/B | Heterozygous A- |  |  |
|  | 202A, 202A/ 376G, 376G | A-/A- | Homozygous A- |  |  |

** Response from authors it was good suggestion and added.

- Table 6:
  - It is unclear why different categories are presented in this table compared with figure 4 – it would be easier to compare if same groupings were used in both.
  - It is unclear what ‘all other’ refers to here – are these individuals that **do** have a variant at 376/202 (this table title suggests only variant individuals are included) but its unclear which specific mutation is present?
  - Again, I don’t think the mean is necessary here, id suggest the authors only report the median.
  - It may be beneficial to add here ‘translations’ of the genotypes for easier interpretation, e.g. a column that reads: ‘B’ for normal males, ‘A/A’ for homozygous A females etc.
- Figures 1-3: I suggest adding units to the x axis – U/g Hb.
- Figure 4: It is unclear what ‘other’ refers to here, perhaps a footnote explaining this would be useful. Are these individuals who could not be genotyped at these loci?

*Minor typographical/textual Comments:*

- Title – Likely a typo, but I would recommend adding a space between phosphate and dehydrogenase in the title.
- Abstract: ‘Such evidence based data will guide the expansion of primaquine for P. vivax infections radical cure.’ Consider changing to ‘… radical cure of *P. vivax* infections’.

** Response from authors All these changes have been incorporated.

Reviewer #1: The spectrophotometry procedures require further details:
Was venous or capillary blood used?

How were samples stored prior to spectrophotometry?

What spectrophotometry assay was used and at what temperature?

 Specify the type of spectrophotometer used
 How was Hb measured?

** Response from authors All these changes have been incorporated.

Reviewer #1: I am concerned about the findings from the reference method spectrophotometry. The article presents G6PD activity ranging from 0.6U/gHb to 37.7U/gHb, the latter is higher than has been reported before in humans. The distribution of activities for males should show a bimodal distribution and this is not the case, in contrast high proportion of males show an intermediate G6PD activity, which typically is associated with heterozygous females only. Interestingly the proportion of females with less than 30% G6PD activity and typically associated with homozygosity is higher than for males, again this has not been described to date and is quite unlikely. Finally, the Carestart RDT identified 14% of all samples as G6PD deficient, while only 4% were deficient by spectrophotometry. A meta-analysis on the Carestart RDT (Performance of the Access Bio/CareStart rapid diagnostic test for the detection of glucose-6-phosphate dehydrogenase deficiency: A systematic review and
meta-analysis. PLoS Med) found sensitivity and specificity to be above 95%, significantly higher than the reported specificity in this article. This seems to suggest substantial issues with spectrophotometry and question many of the main findings. Can the authors discuss this in the discussion?

** Response from authors: all analyses have been extensively revised and fully updated from source data; we have added discussion about potential limitations of the kit utilised to address these issues.

Were correct statistical analysis used to support conclusions?

  I assume that what the authors call the trimmed male median is better known as the adjusted male median (if correct, kindly stick to the latter term which is much more broadly used). It would be excellent if the authors in addition to a 30% threshold could add a 70% threshold to their considerations. The latter is highly relevant for the future introduction of tafenoquine and to identify heterozygous females. Can the authors provide more details in the statistics section? Rather than stating that continuous data were compared “using non-parametric median tests”, name them. The authors next state that categorical variables were compared using the Fishers exact test, however for larger samples a Chi2 test is more appropriate, for paired samples as would be expected from categorized results from spec vs. the RDT the McNemars test for correlated proportions is more suitable.

** Response from authors: all required changes have been incorporated.

Reviewer #1: Parts of the manuscript need major revision for spelling, grammar, clarity and consistency.

** Response from authors: major revisions were done.

Reviewer #2: The final number of G6PD deficient cases do not support an association to weight claimed by the authors, but this is called out in my feedback to the authors

Reviewer #2: The diagnostic performance analysis is not complete and the contingency tables are either incorrect, the sensitivity is wrong
Tables can be made clearer.

Editorial and Data Presentation Modifications?
•     Parts of the manuscript need major revision for spelling, grammar, clarity and consistency.

** Response from authors: we have revised language and corrected typos and inconsistencies.

Reviewer #2: The authors present an interesting study assessing the G6PD prevalence in two locations in Sudan, as well as conduct an evaluation of the CareStart G6PD test. Below list some minor and some significant topics that should be addressed.
Minor:
1.     Page 6 under “Study Areas”  February should be spelled correctly
2.     Page 6: “The gold standard biochemical G6PD measurement was used according to a previously published methods”  the authors should provide more details: for example were reagent developed internally, were they purchased, if so what kit was used?
3.     Figures 1,2,and 3, the male distributions do not show clear bimodal patterns as would be expected. The authors should spend some time to explain this. How long were specimens shipped? Is there a chance there was some specimen integrity issues?
4.     Top of page 7 “The two tests were performed by two independent technicians who were blinded to each one another results.” This sentence needs clarification: were two tests conducted per individual?
5.     Page 11:” AB variant was present in 1.1% (XX/557) of study subjects” XX needs to be replaced with a number
6.     Page 11. Discussion: Paragraph starting “It should be emphasized that Sudan was highlighted as an area with unusually large uncertainty in the geostatistical models…” this paragraph does not seem to add anything to the discussions and should be removed.

Major
1.     Table 4. Either the rows are mislabelled or the sensitivity of the CareStart is only 18% (4/22)
2.     The authors should provide full performance indicators, sensitivity, specificity  NPV and PPV
3.     Results and Discussion regarding genotyping. The description of the genotyping is confusing, it is not clear from the genotyping what proportion of the G6PDd (n=22) were confirmed to be genotypically deficient.
4.     Page 11, Discussion. Association to weight. The authors can should remove or reduce the importance of this association in their abstract, and discussion, based on the fact that this is based on 22 deficient samples and the statistical significance is weak. This is shown by the very useful set of other indicators they collected which should based on literature on humans (not mice!) have stronger associations to G6PD deficiency.
5.     Discussion: genotyping “The most common G6PD genetic variant carried by subjects tested in this study is associated with a normal to moderate G6PD enzyme activity in whom drug-induced hemolysis may be self-limiting” This conclusion is a little misleading, world wide the majority of G6PD variants are normal to mild since the prevalence of G6PDd is rarely above 1%. As the authors state a limitation of this study is that the more severe G6PD variants were not investigated in this study, and so their presence cannot be excluded.

** Response from authors All these changes have been incorporated.
